# Case report: Malignant priapism: penile metastasis from prostate cancer with low serum PSA level

**DOI:** 10.3389/fonc.2024.1395301

**Published:** 2025-01-10

**Authors:** Zhiqiang Zhang, Mengfan Xu, Muhan Shang, Zhiqi Liu, Lei Yang, Dexin Yu

**Affiliations:** Department of Urology, Second Affiliated Hospital of Anhui Medical University, Hefei, Anhui, China

**Keywords:** prostate cancer, prostate-specific antigen, priapism, penectomy, positron emission tomography/computed tomography

## Abstract

**Background:**

Penile metastasis originating from prostate cancer is an extremely rare condition, typically associated with a poor prognosis. Therapeutic approaches are not well established and may require individualized adaptation based on clinical assessment. Radiotherapy is commonly utilized to alleviate symptoms. For patients presenting with priapism, palliative penectomy is often recommended.

**Case presentation:**

This report describes a case of penile metastasis from prostate cancer in a 74-year-old man who presented with priapism. Positron emission tomography/computed tomography (PET/CT) imaging identified metastases in the penis, along with multiple metastatic sites in the lungs, left iliac vascular lymph nodes, abdominal and pelvic lymph nodes, and bones. A palliative penectomy was performed to relieve symptoms, and postoperative pathology confirmed the presence of penile metastasis originating from prostate cancer. Following the penectomy, the patient received ongoing androgen deprivation therapy (ADT) along with androgen receptor antagonists (enzalutamide).

**Conclusions:**

Penile metastasis from prostate cancer is a rare condition and is often initially misdiagnosed due to the presentation of occult malignancy. This case highlights the need for clinicians to enhance their understanding and diagnostic accuracy regarding penile metastases. Imaging techniques such as Fluorine-18-fluorodeoxyglucose positron emission tomography/computed tomography (18F-FDG PET/CT) and Gallium-68 prostate-specific membrane antigen positron emission tomography/computed tomography (Ga-68 PSMA PET/CT) can detect prostate cancer lesions even at low serum prostate-specific antigen (PSA) levels, thereby improving diagnostic precision for prostate cancer.

## Background

Prostate cancer is one of the most common cancer in men; however, metastasis to the penis is exceedingly rare, with an incidence rate between 0.3% and 0.5% (1). Clinical manifestations are varied, presenting as palpable nodules or skin changes on the penis (with or without ulceration) and may also include urinary symptoms such as urinary retention or dysuria (1–3). Although the penis has a complex vascular network interconnected with pelvic organs, metastasis of prostate cancer to the penis is uncommon, likely due to specific anatomical factors influencing metastatic pathways, including direct invasion, implantation, retrograde venous flow, and arterial spread. The prognosis for penile metastasis of prostate cancer remains generally poor (2), and therapeutic strategies are not well established.

## Case report

A 76-year-old man with a history of obstructive lower urinary tract symptoms, presenting an initial prostate-specific antigen (PSA) level of 3.56 ng/mL, underwent transurethral resection of the prostate (TURP). Histological examination of the tissue obtained from TURP confirmed prostate cancer, with a Gleason score of 5 + 4 = 9. Given the patient’s preference to avoid surgery and radiotherapy, androgen deprivation therapy (ADT) was initiated with bicalutamide and leuprorelin. However, 5 months post-TURP, he developed a complex urethral stricture accompanied by a low serum PSA level (PSA <0.2 mg/L). Cystourethroscopic evaluation via ureterorenoscopy revealed no signs of malignant invasion into the urethra or bladder, and he was subsequently managed with a vesicostomy. Six months following TURP, he reported worsening penile pain and a persistent erection without cutaneous nodules or ulceration. His visual analog scale (VAS) pain score was recorded at 4 (range, 0–10), and celecoxib was administered twice daily over 1 month. Lung computed tomography (CT) revealed multiple pulmonary nodules of undetermined cause, while a pelvic CT scan showed an enlarged prostate without lymph node enlargement or soft-tissue masses. A digital rectal examination indicated a hard, enlarged prostate with a firm, nodular texture. Because of a slight increase in PSA level (0.233 ng/mL), abiraterone combined with prednisone was subsequently administered.

At 10 months after TURP, laboratory tests indicated a hemoglobin level of 11.5 g/dL, a total leukocyte count of 11.35 × 10^9^/L, a platelet count of 302 × 10^9^/L, and a sterile urine culture. The PSA level was 0.273 mg/L. Intracavernous blood gas analysis revealed hypoxic, low-flow priapism, with a pH of 6.9, a PO2 of 6.2 mmHg, and a PCO2 of 84 mmHg, leading to a diagnosis of ischemic priapism. The patient received intravenous antibiotics (fosfomycin, 4.0 g every 8 h) for infection control, along with celecoxib (100 mg twice daily) to manage penile pain for a duration of 1 week. However, there was no significant improvement in priapism or penile pain symptoms.

**Figure 1:**
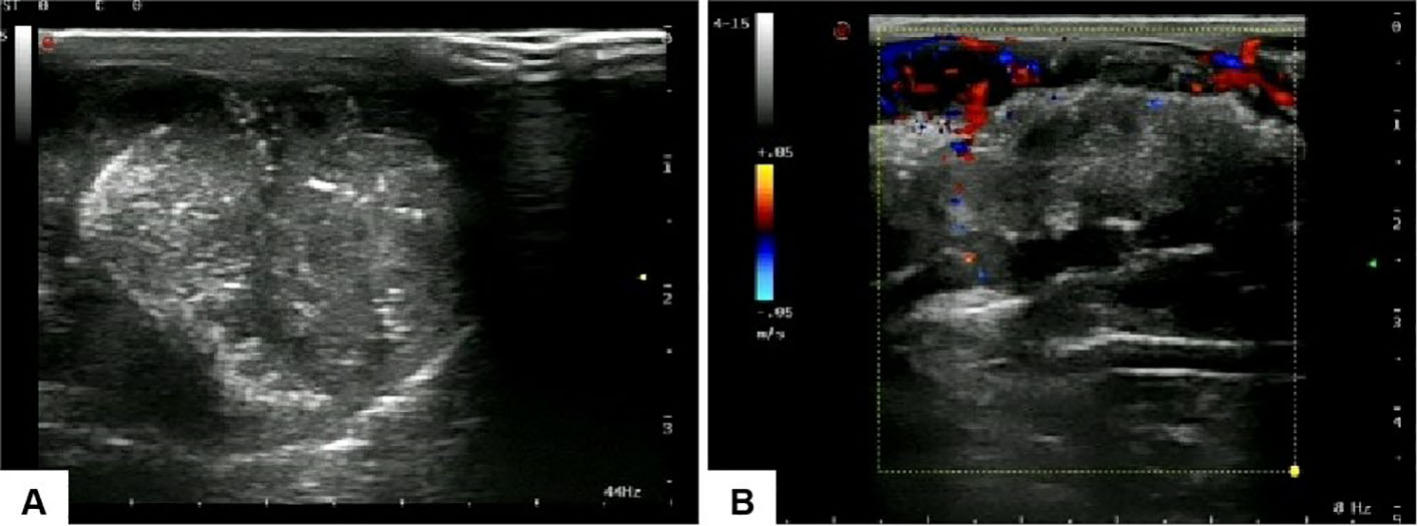
Ultrasound images of the penis: **(A)** clusters of calcified lesions are visible within the penile structure, and **(B)** fascicular blood flow signals are observed.

**Figure 2:**
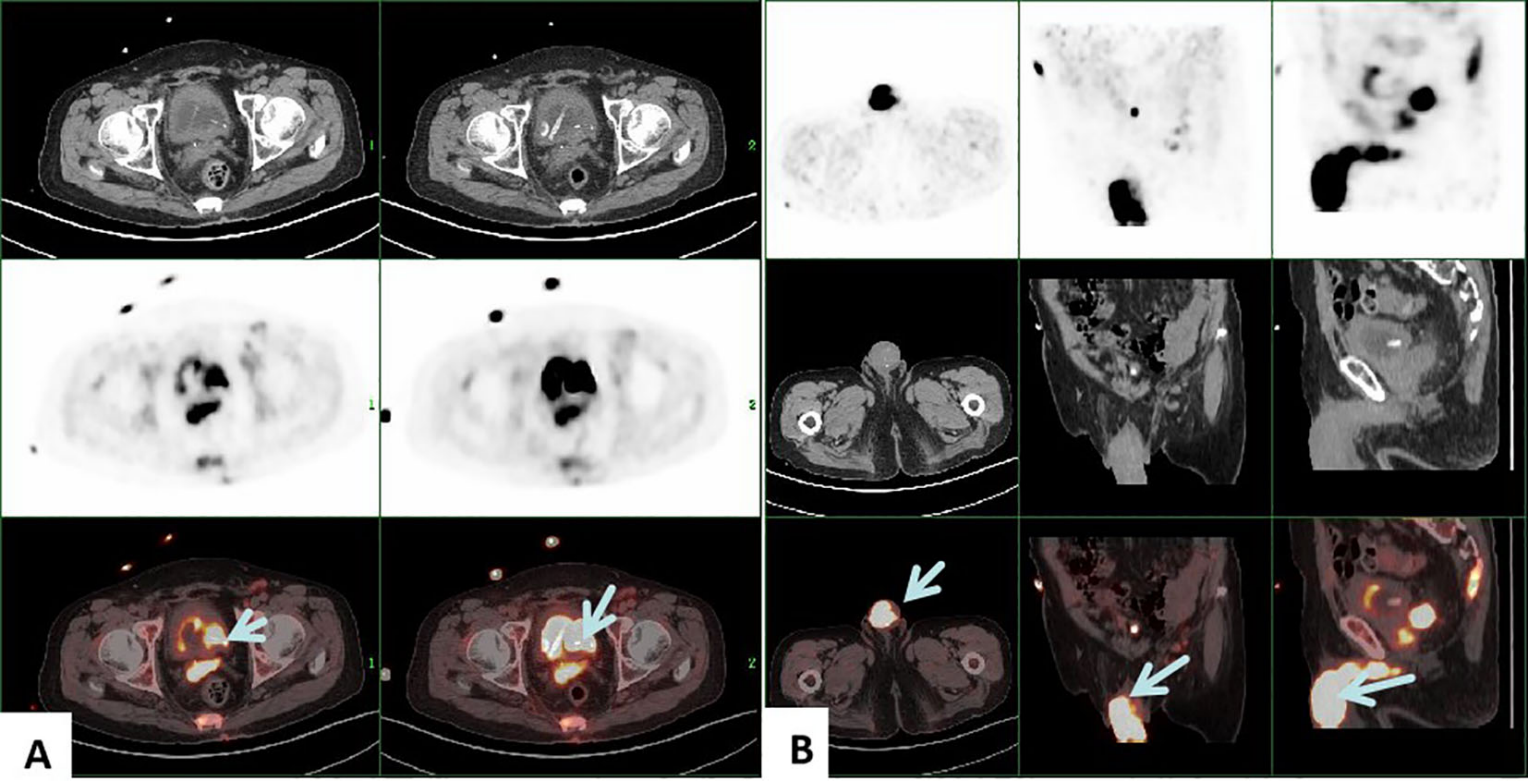
PET/CT revealed that the higher uptake of prostate gland (SUVmax 15.3) **(A)** and penile lesions (SUVmax 18.7) **(B)**, indicating local recurrence and penile metastasis from prostatic cancer.

**Figure 3:**
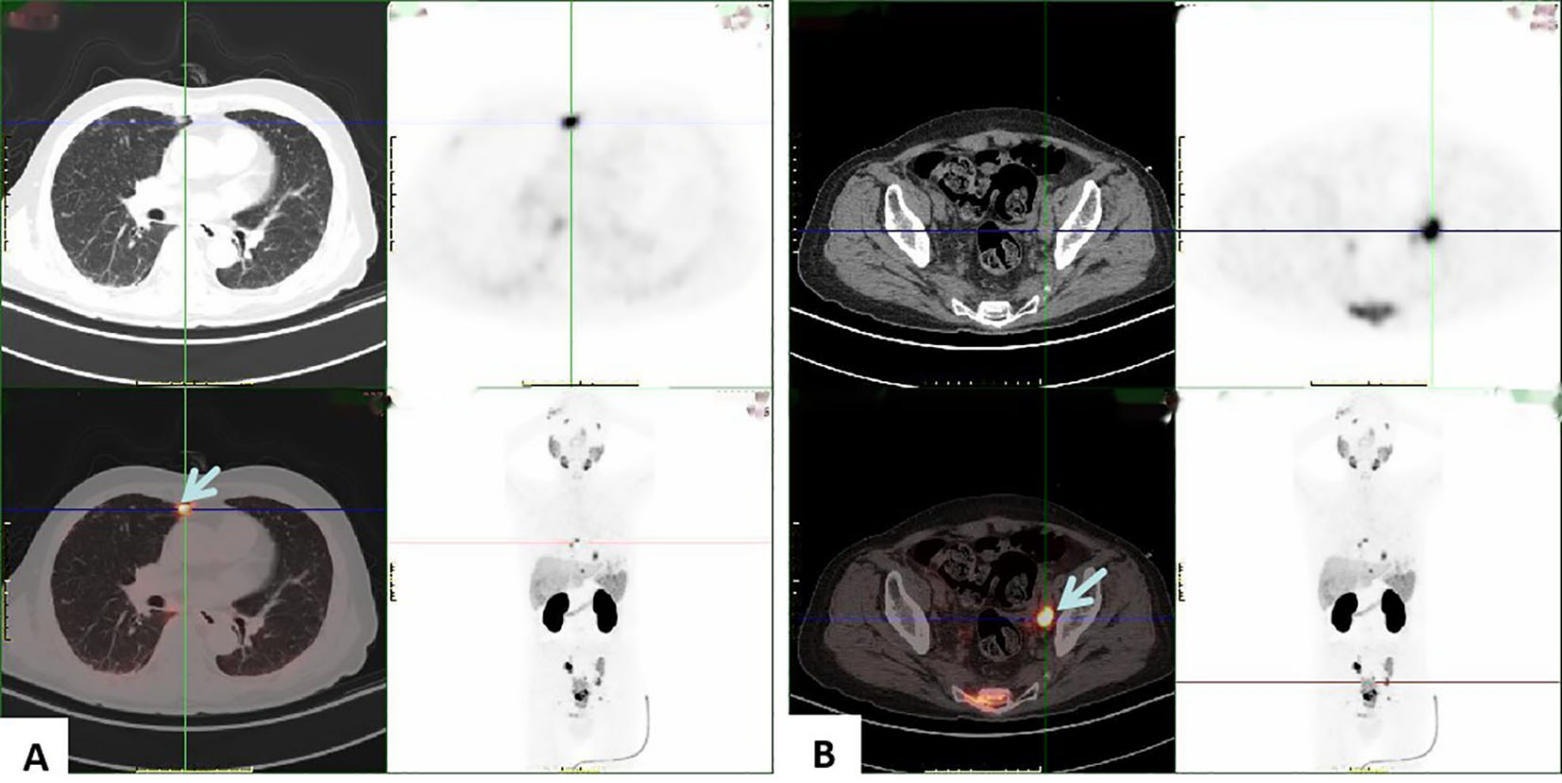
PSMA PET/CT shows high uptake in the lungs **(A)** and left iliac vascular lymph nodes **(B)**, suggesting metastasis to the lungs and left iliac vascular lymph nodes from prostatic cancer.

**Figure 4:**
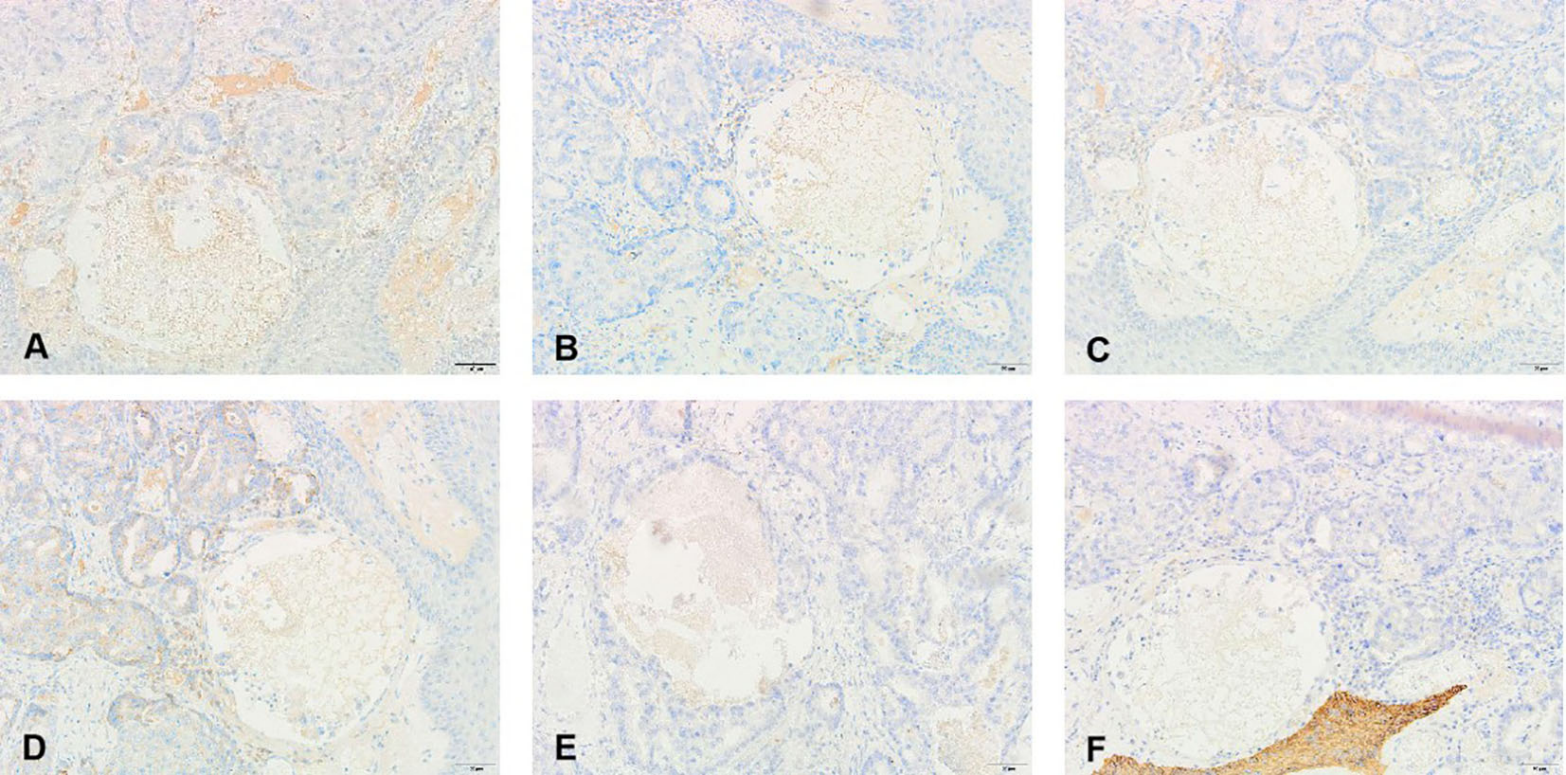
Pathological and immunohistochemical findings of the metastatic penile tumor: **(A)** prostate-specific antigen(+), **(D)** alpha-methylacyl-CoA racemase (P504S)(+), **(B)** Chromogranin A (CgA)(−), **(C)** alpha-Syn (Syn)(−), **(E)** cytokeratin 7 (CK7)(−), and **(F)** cytokeratin 56 (CK7)(−).

Penile ultrasonography revealed no nodules in the corpora cavernosa or corpus spongiosum. However, the ultrasound image displayed clusters of calcified lesions (**Figure 1A**) and fascicular blood flow signals (**Figure 1B**). A follow-up chest CT scan performed 10 months later showed multiple lung metastases. The ^18^F-FDG PET/CT scan indicated significant uptake in penile lesions (SUVmax 18.7) (**Figure 2A**), an enhancing lesion in the prostate gland (SUVmax 15.3) (**Figure 2B**), as well as uptake in the liver (SUVmax 5.32) and lungs (SUVmax 12.1), alongside bone metastases. Given the suspicion of malignancy, a penile puncture biopsy was recommended. However, because of penile pain and the presence of a vesicostomy, the patient declined the biopsy and palliative chemotherapy. Following detailed discussions with the patient and family, a palliative penectomy was performed to alleviate penile pain. Ga-68 PSMA PET/CT post-penectomy revealed metastases in the lungs (**Figure 3A**), left iliac vascular lymph nodes (**Figure 3B**), abdominal and pelvic nodes, and multiple bones (ilium, sacrum, coccyx, acetabulum, pubis, and ischium), as well as local recurrence of prostate cancer. Postoperative pathological analysis of penile tissue confirmed prostate adenocarcinoma with a Gleason score of 9 (4 + 5). Immunohistochemistry results (**Figure 4**) showed negative staining for CK5/6, Syn, CgA, CK7, CD56, and Villin, with positive staining for PSA and P504S, definitively indicating penile metastasis of prostate cancer. In the context of castration-resistant prostate cancer, the patient was subsequently maintained on continuous ADT and androgen receptor antagonists (enzalutamide) following the palliative penectomy.

At the last follow-up (5 months post-penectomy), the patient had completed five cycles of enzalutamide, achieving a PSA level of <0.008 ng/mL, and reported favorable progress. He expressed satisfaction with pain relief, as his VAS score had decreased to 0–1 points. A timeline detailing PSA progression, clinical course, and treatment is available in the **Supplementary Figure S1**.

## Discussion

Penile metastases are rare occurrences, typically secondary to other genitourinary cancers, with prostate cancer metastasis to the penis representing an incidence of only 0.3% to 0.5% (1). Such metastases generally emerge as a late-stage clinical manifestation in the natural course of metastatic prostate cancer and are often associated with a poor prognosis (2). Clinically, penile metastases may present with painless nodules, surface nodules, ulceration, erythema, urinary retention, irritative urinary symptoms, perineal pain, hematuria, and priapism (3). In this case, priapism occurred without the presence of cutaneous nodules or ulceration.

Priapism is defined as a prolonged penile erection lasting over 4 h in the absence of sexual stimulation (4). Despite the rich vascularization of the penis, penile metastases remain exceedingly rare, with malignant priapism more commonly associated with hematologic malignancies, such as myeloid or lymphoblastic leukemia, due to blood hyperviscosity (5). Malignant priapism resulting from solid tumors is very rare and was first documented in 1870 (6), often indicating a poor prognosis. Priapism is categorized into ischemic, non-ischemic, and stuttering forms (7), with most cases of malignant priapism secondary to solid tumors being ischemic (7). Tumor invasion into lymphovascular structures, including arteries, veins, and lymphatics, can cause complete vascular blockage, resulting in prolonged and persistent erection (8).

Owing to its rare clinical presentation, this case was initially misdiagnosed as penile sclerosis (2). A preoperative physical examination did not indicate signs of prostate cancer. Rapid progression and metastasis of prostate cancer without elevated PSA levels is uncommon; in this case, serum PSA remained stable post-TURP, with the natural progression to distant metastases being observed. Unlike other cases documented in the literature (2, 3), this case presented with priapism without accompanying cutaneous nodules or ulceration.

Radiologic imaging plays a critical role in enhancing diagnostic precision. Color Doppler sonography typically reveals hypoechoic or heterogeneous nodules in the corpora cavernosa and/or corpus spongiosum (9); however, in this case, ultrasound showed no evidence of penile metastasis. Pelvic MRI, which provides high contrast with soft tissues, is useful for assessing prostate cancer and related metastatic lesions (6). Ga-68 PSMA PET/CT has become widely used for staging and re-staging prostate cancer, recommended as a reliable modality for early differentiation of penile metastatic lesions originating from prostate cancer (10). Notably, Ga-68 PSMA PET/CT is capable of detecting prostate cancer lesions even at low serum PSA levels (11). Kamaleshwaran et al. documented the first instance of Ga-68 PSMA PET/CT identifying penile metastasis from prostate cancer associated with malignant priapism (8).

The differential diagnosis for this condition includes idiopathic priapism, venereal or infectious diseases, tuberculosis, chancroid lesions, Peyronie’s disease, syphilis, and primary penile tumors (12, 13). Histological diagnosis, typically achieved through local biopsy methods such as fine-needle aspiration cytology or excision biopsy, has enhanced diagnostic accuracy. However, a consensus on treatment approaches remains undeveloped, as therapy may need to be tailored to each patient based on clinical judgment, disease extent, and patient condition. Radiotherapy is commonly employed to relieve symptoms. Palliative penectomy is an option for treating patients with penile metastases from prostate cancer, particularly in cases of malignant priapism, as it can provide lasting symptom relief (14, 15). Only a limited number of cases in the literature report PSMA-PET/CT detection of penile metastasis from prostate cancer (8, 11, 16); none of these cases involved TURP. In this case, however, clinical progression and castration resistance emerged over time during ADT following TURP. Similar to previous cases (16, 17), progression post-TURP with a low serum PSA level can be easily overlooked during treatment and follow-up. PSMA PET/CT can aid in the early detection of metastatic lesions from prostate cancer, which may improve clinical decision-making, particularly in cases with low serum PSA levels.

The prognosis for penile metastases is generally poor, with an average survival duration of about 9 months (8), underscoring the need for treatments focused on symptom relief. Cases of penile metastases presenting with priapism are associated with a significantly worse prognosis compared to those without priapism (18). Unlike prior reports, the current case involved ongoing ADT, androgen receptor antagonists (enzalutamide), and palliative penectomy, resulting in a survival duration exceeding 12 months. The combination of the next-generation androgen receptor pathway inhibitor (enzalutamide) with palliative penectomy may extend survival for patients with penile metastasis from prostate cancer.

In conclusion, penile metastasis from prostate cancer is extremely rare and may manifest through a range of symptoms. Ga-68 PSMA PET/CT scanning is a valuable diagnostic tool for detecting metastatic lesions associated with prostate cancer. Given the typically poor prognosis for penile metastases, palliative care combined with systemic treatment is recommended to enhance the patient’s quality of life.

## Data Availability

The original contributions presented in the study are included in the article/**Supplementary Material**. Further inquiries can be directed to the corresponding author.

## Supplementary material

Supplementary Figure S1 Timeline of PSA progression, clinical course, and treatment interventions (black-ADT, yellow-Abiraterone plus prednisone, red-Enzalutamide; TPSA: ng/mL).

## References

[1] Martz N Benziane-Ouaritini N Gautier M Brenot-Rossi I Montagne L Salem N. Brachytherapy for oligometastatic prostate cancer to the penis. J Contemp Brachyther. (2021) 13:593–7. doi: 10.5114/jcb.2021.109754 PMC8565627 34759985

[2] Dai Y Shi BL Zhang J Liu SN Jia YT. Penile metastasis from prostate cancer misdiagnosed as Peyronie disease: a case report. Sex Med. (2023) 11:qfac011. doi: 10.1093/sexmed/qfac011 37007855 PMC10065180

[3] Philip J Mathew J. Penile metastasis of prostatic adenocarcinoma: Report of two cases and review of literature. World J Surg Oncol. (2003) 1:16. doi: 10.1186/1477-7819-1-16 14521716 PMC212152

[4] Broderick GA Kadioglu A Bivalacqua TJ Ghanem H Nehra A Shamloul R. Priapism: pathogenesis, epidemiology, and management. J Sex Med. (2010) 7:476–500. doi: 10.1111/j.1743-6109.2009.01625.x 20092449

[5] Ralph O Shroff N Johnson MJ AlNajjar HM Ralph D. Malignancy: A rare, important and poorly understood cause of priapism. Sex Med Rev. (2021) 9:312–9. doi: 10.1016/j.sxmr.2019.11.002 31902677

[6] Fiaschetti V Liberto V Claroni G Loreni G Formica V Roselli M. Relevance of computed tomography and magnetic resonance imaging for penile metastasis after prostatectomy: uncommon case report and brief review of the literature. Radiol Case Rep. (2016) 11:255–9. doi: 10.1016/j.radcr.2016.04.003 PMC4996907 27594962

[7] Shigehara K Namiki M. Clinical management of priapism: A review. World J Mens Health. (2016) 34:1–8. doi: 10.5534/wjmh.2016.34.1.1 27169123 PMC4853765

[8] Kamaleshwaran KK Balasundararaj BKP Jose R Shinto AS. Penile metastasis from prostate cancer presenting as Malignant priapism detected using gallium-68 prostate-specific membrane antigen positron emission tomography/computed tomography. Indian J Nucl Med. (2018) 33:57–8. doi: 10.4103/ijnm.IJNM_107_17 PMC5798101 29430118

[9] Nakayama F Sheth S Caskey CI Hamper UM. Penile metastasis from prostate cancer: diagnosis with sonography. J Ultrasound Med. (1997) 16:751–3. doi: 10.7863/jum.1997.16.11.751 9360239

[10] Tatkovic A McBean R Schoeman J Wong D. Prostate penile metastasis: Incidence and imaging pattern on (68) Ga-PSMA PET/CT. J Med Imaging Radiat Oncol. (2020) 64:499–504. doi: 10.1111/1754-9485.13052 32449823

[11] Maurer T Eiber M Schwaiger M Gschwend JE. Current use of PSMA-PET in prostate cancer management. Nat Rev Urol. (2016) 13:226–35. doi: 10.1038/nrurol.2016.26 26902337

[12] Cardoso Guimaraes G Rodrigues De Souza R Paiva Gadelha Guimaraes A Filho WD Valeschka De Matos Granja N Karan Kalil R. Penile metastasis of chondrosarcoma of the jaw. Urology. (2003) 61:837. doi: 10.1016/S0090-4295(02)02431-7 12670583

[13] Ansari H Prashant R Franks A. Prostatic carcinoma metastasis to the penis–an uncommon site. Lancet Oncol. (2003) 4:705–6. doi: 10.1016/S1470-2045(03)01251-8 14602253

[14] Landen L Devos G Joniau S Albersen M. Penile metastasis in prostate cancer patients: Two case reports, surgical excision technique, and literature review. Curr Urol. (2023) 17:165–72. doi: 10.1097/CU9.0000000000000093 PMC10337815 37448616

[15] Tabei SS Baas W Brooks A Kim EH Smith Z Murphy GP. Malignant priapism: case report and update on management protocols. Transl Androl Urol. (2023) 12:1607–13. doi: 10.21037/tau-23-327 PMC10643380 37969781

[16] Li Y Li Y Dong S Chen J Yang P Li J. Case report: 18F-PSMA-1007 PET/CT avid solitary penile metastasis of castration-resistant prostate cancer with a PSA of 0.072 ng/ml. Front Oncol. (2022) 12:881896. doi: 10.3389/fonc.2022.881896 35530336 PMC9067612

[17] He D Zeng J Li X Wu K Wu D He H. Priapism as the initial manifestation of a penile and lower limb cutaneous metastasis of prostate adenocarcinoma with low serum PSA level. J Androl. (2012) 33:1160–4. doi: 10.2164/jandrol.112.016873 22700763

[18] Cocci A Hakenberg OW Cai T Nesi G Livi L Detti B. Prognosis of men with penile metastasis and Malignant priapism: a systematic review. Oncotarget. (2018) 9:2923–30. doi: 10.18632/oncotarget.23366 PMC5788693 29416825

