# Supplementary figures and images for: Case report: Malignant priapism: penile metastasis from prostate cancer with low serum PSA level
Source: Front Oncol. 2025 Jan 10;14:1395301. doi: 10.3389/fonc.2024.1395301 (PMC11757117; doi:10.3389/fonc.2024.1395301)

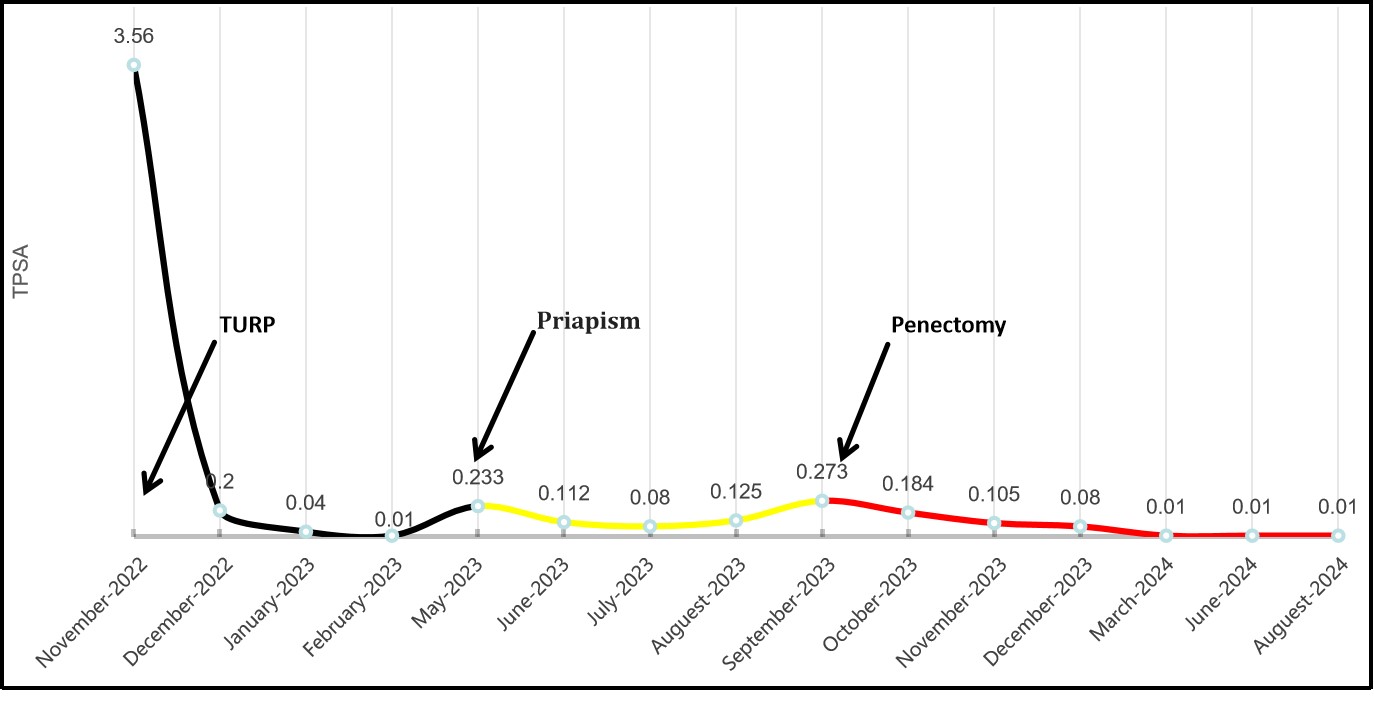

Supplement: Supplementary Figure S1 — Timeline of PSA progression, clinical course, and treatment interventions (black-ADT, yellow-Abiraterone plus prednisone, red-Enzalutamide; TPSA: ng/mL). [file Image1.jpeg]
